# Effectiveness of early part-time sick leave in musculoskeletal disorders

**DOI:** 10.1186/1471-2474-9-23

**Published:** 2008-02-25

**Authors:** Kari-Pekka Martimo, Leena Kaila-Kangas, Johanna Kausto, Esa-Pekka Takala, Ritva Ketola, Hilkka Riihimäki, Ritva Luukkonen, Jaro Karppinen, Helena Miranda, Eira Viikari-Juntura

**Affiliations:** 1 Team of Musculoskeletal Disorders, Centre of Expertise of Health and Work Ability, Finnish Institute of Occupational Health, Topeliuksenkatu 41 a A, 00250 Helsinki, Finland; 2 Centre of Expertise of Health and Work Ability, Finnish Institute of Occupational Health, Topeliuksenkatu 41 a A, 00250 Helsinki, Finland; 3 Team of Statistical Services, Centre of Expertise of Good Practices and Competence, Finnish Institute of Occupational Health, Topeliuksenkatu 41 a A, 00250 Helsinki, Finland

## Abstract

**Background:**

The importance of staying active instead of bed rest has been acknowledged in the management of musculoskeletal disorders (MSDs). This emphasizes the potential benefits of adjusting work to fit the employee's remaining work ability. Despite part-time sick leave being an official option in many countries, its effectiveness has not been studied yet. We have designed a randomized controlled study to assess the health effects of early part-time sick leave compared to conventional full-day sick leave. Our hypothesis is that if work time is temporarily reduced and work load adjusted at the early stages of disability, employees with MSDs will have less disability days and faster return to regular work duties than employees on a conventional sick leave.

**Methods/Design:**

The study population will consist of 600 employees, who seek medical advice from an occupational physician due to musculoskeletal pain. The inclusion requires that they have not been on a sick leave for longer than 14 days prior to the visit. Based on the physician's judgement, the severity of the symptoms must indicate a need for conventional sick leave, but the employee is considered to be able to work part-time without any additional risk. Half of the employees are randomly allocated to part-time sick leave group and their work time is reduced by 40–60%, whereas in the control group work load is totally eliminated with conventional sick leave. The main outcomes are the number of days from the initial visit to return to regular work activities, and the total number of sick leave days during 12 and 24 months of follow-up. The costs and benefits as well as the feasibility of early part-time sick leave will also be evaluated.

**Conclusion:**

This is the first randomised trial to our knowledge on the effectiveness of early part-time sick leave compared to conventional full-time sick leave in the management of MSDs. The data collection continues until 2011, but preliminary results on the feasibility of part-time sick leave will be available already in 2008. The increased knowledge will assist in better decision making process regarding the management of disability related to MSDs.

**Trial Registration:**

International Standard Randomised Controlled Trial Number Register, register number ISRCTN30911719

## Background

Evidence is increasing on the importance of staying active instead of bed rest in the management of musculoskeletal disorders (MSDs) and related disability [1-4]. It has been recommended that total absence from work due to non-specific back pain should be avoided because of its possible delaying effects on recovery [5]. In reducing absence from work due to MSDs or other pain-related conditions, workplace-based return-to-work interventions have shown to be effective [6]. The evidence is particularly strong for offers to accommodate work during recovery and contacts between healthcare provider and the workplace.

In a Finnish survey, employees with health problems were asked to assess their ability to work prior to visiting an occupational health (OH) physician [7]. The results showed that employees with MSDs report partial work ability almost twice as often as complete inability to work (28% vs. 16%). In the same survey, both employees with MSDs and their physicians often regarded work-related interventions as potentially beneficial to improve recovery. The importance of these interventions is further supported by the evidence on the significance of work-related factors in the aetiology of MSDs [8].

Employees with partial work ability could be encouraged to stay at work instead of taking sick leave. It requires, however, flexible systems to adjust work load in order to accommodate the employee until the disability has improved. Part-time sick leave system in Finland enables the employee to work 40–60% of the daily working time but only after the full-time sick leave has lasted for almost three months. In most cases, part-time sick leave is economically more rewarding than total absence from work since, in addition to the salary for the worked hours, the Social Insurance Institution provides the employee a monetary compensation, which is half of that for full-time sick leave. So far, part-time sick leave has been used, however, less than expected.

In some other countries (e.g., Sweden, Norway, Denmark), taking part-time sick leave is allowed without any preceding full-time sickness absence. Yet, the effectiveness of part-time sick leave has been poorly studied. A Norwegian cluster-randomised study on "active sick leave" (return to adjusted work supported by social security after conventional sick leave had lasted 16 days or more) showed no beneficial effects, partly because part-time sick leave system was so seldom used [9]. Users' contentment seems to be, however, high; 92% of employees on part-time sick leave in a Swedish survey were satisfied with the arrangement [10]. Two-thirds of those on full-time sick leave considered part-time sick leave as a potentially good alternative for them. Yet, some disadvantages have also been detected: a Swedish study with a follow-up of 1.5 years found that part-time sick leaves tended to last longer than conventional sick leaves [11]. The authors concluded that the effectiveness of part-time sick leave should be studied in a randomized setting.

This randomized controlled study was designed to assess the health-related and economical effects of early part-time sick-leave compared to conventional sick leave in employees with MSDs.

## Methods/Design

### Identification and eligibility of study participants

**Figure 1:**
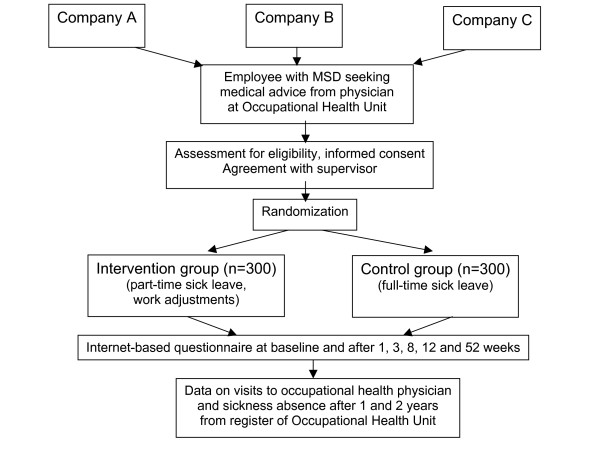
Study design.

The study will be performed in several OH units of medium sized and large private or public enterprises, in which the employees are known to be exposed to physically strenuous or static work tasks (Figure 1). Employees who seek medical advice primarily due to musculoskeletal pain in the neck or shoulder region, back, or upper or lower extremities are eligible to the study. The symptoms and related disability must warrant prescription of full-time sick leave according to the current practice, but the physician considers the employee to be able to work part-time without the risk of the health condition to deteriorate. Table 1 shows the inclusion and exclusion criteria.

**Table 1:** Eligibility of the participants

**Inclusion criteria**
- 18 to 60 years of age
- permanent or long-term employment (30 hours or more per week)
- no sick leave or other absence exceeding two weeks during the preceding month
- not more than 30 days on sick leave due to any health problem during three preceding months
- employee is not listed for any surgery that requires more than one week of sickness absence
- no plan for longer absence from work during 12 months after enrollment
- an employee can be enrolled only once
**General exclusion criteria**
- acute infections
- major accidental injury
- active inflammatory arthritis
- suspected occupational injury or disease
- malignant tumour diagnosed or treated during the preceding year
- severe mental disorder
- pregnancy
- severe pain (> 7 on a scale from 0 to 10)
- pain interferes with sleep severely (> 7 on scale from 0 to 10)
**Pain area specific exclusion criteria**
Back region:
- muscle weakness in the lower extremities related to back pain
- positive straight-leg-raising test
- pain-related trunk list
- painful spasm of the back when bending forward
Neck and shoulder region:
- muscle weakness in the upper extremities related to the pain
- head compression or movements induce radiating pain below elbow level
- painful torticollis
Shoulder and upper extremity regions:
- muscle weakness related to the pain
- severe pain in movements interfering with most functions
Lower extremities:
- pain prevents walking

### Interventions

During the initial visit, the OH physician will inform the employee about the study and its aims. If the employee agrees to participate, informed consent will be signed. It includes a permission to contact the supervisor, preferably during the same visit, in order to verify whether the work-related arrangements for part-time sick leave are feasible, in case the employee is allocated to the intervention group. If the supervisor disagrees, the employee will be excluded from the trial.

Once the agreements from the employee and the supervisor have been obtained and before the randomisation has occurred, the physician will determine the length of the sickness absence based on symptoms (Table 2), clinical findings [12,13] and background information (Table 3). If the employee is allocated to part-time sick leave (intervention group), daily work load will be reduced by restricting work time. Also, if necessary, remaining work tasks will be modified so that working should be possible despite the symptoms. In the control group, work load will be eliminated with full-time sick leave. Both groups will receive appropriate medical advice, and the need for medical treatments and a control visit will be determined as usual.

**Table 2:** Health-related variables inquired at baseline and during follow-up

**Outcome**	**Measure**
Main reason for the visit (at baseline)	Localisation and characteristics of pain
Pain intensity	Scale from 0 to 10
Interference with work activities	Scale from 0 to 10
Interference with sleeping	Scale from 0 to 10
Self-assessed functioning	Oswestry Back questionnaire [15] Quick DASH (Disabilities of the Arm, Shoulder and Hand) [16] COAT (Comprehensive Osteoarthritis Test) for lower extremities [17]
Productivity at work	Quantity and Quality of work [26]
Adjustments at work (during follow-up)	Description of change in physical work load (structured and open questions)

**Table 3:** Explanatory variables measured at baseline

**Assessments by physician**	
Physical examination	Standardised protocol [12, 13]
Weight	Self-reported
Height	Self-reported
**Health-related background information**	
Diabetes, cardiovascular diseases	Interview by physician
Previous sick leaves	OH data base
Depression	Questionnaire [20]
Quality of life	EQ-5D [21]
**Physical work load factors**	
Handling of loads, repetitive movements, use of hand tools, awkward postures at work	Questionnaire [18, 19]
**Background variables**	
Company	Questionnaire
Occupation	
Main work tasks	
Education	
Job seniority	
Work hours	
Commuting time	
Age	
Gender	
Marital status	
Smoking	
Alcohol consumption	
Physical exercise	
Psychosocial risk factors	Adapted from Copenhagen Psychosocial Questionnaire [22]
Fear avoidance	Questionnaire [23]
Procedural and relational justice	Questionnaire [24]
Effort-reward imbalance	Questionnaire [25]

In the following day after the initial visit, those allocated to part-time sick leave will return to work for 40 to 60% of their regular working hours. They are asked to give their supervisor a statement from the physician indicating the duration of part-time sick leave, what kind of work loads are allowed and which are not recommended, and if any additional work adjustments are needed. If the reduction of work time cannot be organised on a daily basis, the employee may work every second day for the whole day and every second day stay at home.

When the sick leave is over, employees in both groups return to regular work. Those who are unable to resume regular work activities will be re-evaluated by an OH physician, who can prescribe either full-time sick leave or continue part-time sick leave, according to the physician's clinical judgement. The length of sick leave will be determined as usual.

If the employee in the part-time sick leave group needs full-time sick leave, part-time sick leave cannot be applied again after full-time sick leave has ended. Return to part-time sick leave is possible in a situation when the health problem relapses within one month after the employee has returned to regular work. The total maximum length of the part-time sick leave is two months. Participation in the study intervention does not affect the employee's right to general social security benefits. Neither does it have any financial effect to the employee, because he or she receives the same compensation (regular salary) during part-time and full-time sick leave. The employer will start receiving compensation after the sick leave has lasted for 10 working days.

### Objectives

Musculoskeletal symptoms typically deteriorate during the work day and towards the end of the work week, and they resolve during break, night rest and weekend [14]. Our hypothesis to be tested in the current study is that employees whose work time is temporarily reduced and work load adjusted during early stage of disability will have less disability days and faster return to regular work duties than employees on conventional sick leave.

### Outcomes

The main outcomes are the number of days from the initial visit to the return to regular work activities and the total number of part- and full-time sick leave days during one- and two-year follow-up. Other outcomes are changes in symptoms as well as in self-assessed functioning during follow-up [15-17].

Data on self-assessed workload will be collected after the initial visit and during part-time sick leave in order to recognise physically strenuous work tasks relevant to MSDs [18,19], as well as to list the work adjustments made during the part-time sick leave. This will be included in an internet based questionnaire together with the symptoms and background variables [20-25]. The questionnaire will be sent to the employees immediately after recruitment and at one, three, eight, 12 and 52 weeks. Those without access to internet will be telephone-interviewed by a researcher. Also, the supervisors in both groups will be interviewed by phone after the employee has returned to work.

Participating employees, their supervisors, OH personnel, human resources management, as well as occupational safety and trade union representatives will be interviewed, and the feasibility of early part-time sick leave will be evaluated based on the qualitative information collected in the interviews. From those employees, who decline to participate, only information on age and gender will be collected. They will also be offered a possibility to provide the main reason for not participating. This can be done with an anonymous questionnaire that can be returned in a closed envelope directly to the researchers. The reason for a negative response from the supervisor is also documented.

The follow-up is extended to two years with the purpose of detecting the possible longer-lasting effects on e.g. illness behaviour. One and two years after the initial visit, the dates and diagnoses of all sickness absences (including those prescribed outside OH services) will be collected from the OH units.

Patient enrolment started in January 2008 and it is planned to last until July 2009. Follow-up of the last participants will finish two years later in 2011. Based on the pilot study, however, preliminary results on the feasibility of early part-time sick leave will be available already in 2008.

### Economic evaluation

Costs and benefits to the employee, employer and society will be estimated in both study groups. Costs due to lost working time will be analysed separately taking into account the compensation to the employer during full- or part-time sick leave. Data on costs of the used health services, medications, and medical aids (due to the main health problem) will also be collected. In addition, the analysis will include the compensation of the lost work input using stand-ins (salary, training time) or overtime (performed by the colleagues of the study subjects), as well as the time the supervisor used for work arrangements.

The non-monetary benefits will be studied based on self-assessed productivity at work [26], as well as the reduction of pain and disability measured on a scale from 0 to 10 (Table 2). If there is a difference between the groups in the outcome measurements, a cost effectiveness analysis will be performed dividing the costs by the units of difference in the outcome. If there is no significant difference between the study groups in any of the health related outcomes, the analysis of total costs in both groups will be applied in making the final conclusions.

### Sample size

A 10% difference between intervention and control group in the proportions of employees returning to regular work at a given time point will be considered significant. The power of the study is aimed at 80%, and the level of significance at 0.05. Assuming a drop-out rate of 10–15%, 600 employees will be needed for the study (300 in both groups). Using previous information on sickness absenteeism in Finnish enterprises, and to ensure a sufficient pool of subjects, we will include in the study base a sufficient number of companies employing up to 30 000 persons.

### Randomisation

Employees will be randomised using sealed opaque envelopes, which contain information on the allocated group. The allocation has been performed at the Finnish Institute of Occupational Health using a random number generator and block randomisation in order to obtain equal size of intervention and control group for the participating physicians.

### Blinding

Due to practical and ethical reasons neither the employee nor the physician will be blinded to group assignment during the initial visit. Allocation is also open to the two interviewers and to the other possible physicians during later visits.

### Statistical methods

A survival analysis will be used to study the time to return to work in the intervention and control group. The amount of sick leave days will be analysed at 12 and 24 months, and the associations between the outcomes and background variables will be analysed using general linear models. In addition, the change in symptoms and disability indices will be studied at various time points using general linear models for repeated measurements.

Subgroup analyses will be performed in relation to patient compliance, type of work, adequacy of work adjustments, previous sick leaves, pain characteristics (such as localisation, duration), and level of disability at baseline. All analyses will be made based on an intention-to-treat principle.

### Ethics

The Coordinating Ethics Committee of Hospital District of Helsinki and Uusimaa has granted approval for this study.

### Clinical Trial Register

This study has been registered at International Standard Randomised Controlled Trial Number Register, register number ISRCTN30911719

## Discussion

The target of this intervention is to adjust work (both work time and demands) to accommodate the disabled employee so that he or she can continue working during recovery from a MSD. As pointed out by Durand et al [27], in this type of intervention work becomes an object of the intervention itself raising various methodological challenges. In addition to the medical judgement by the physician, the intervention requires actions and decisions made by the employee, supervisor, colleagues and employer – each with their own values, objectives, interests, and training [28].

Sickness absence is usually considered as a consequence of a health disorder rather than its treatment and, therefore, in most studies, it has been used as an outcome measure. In this trial, however, the mode of sick leave (part- or full-time) is used as an intervention to affect the outcome, i.e., the quantity of sick leave (number of sick leave days). The potential benefit of the intervention, i.e., the difference in the total number of full- or part-time sick leave days between the intervention and control group, will arise either from the employee's decision to return to regular work earlier than recommended by the physician, or from the need for additional part- or full-time sick leave during the follow-up period.

It is essential in this intervention that the physician determines the length of the disability before allocation, and adheres to this when prescribing either part- or full-time sick leave. This is to avoid bias that might occur if the length of the sick leave is determined differently for part- and full-time sick leave. The risk for bias concerns also the possible control visit, during which the allocation to further part- or full-time sick leave is again open to both the physician and the employee. Inappropriately timed return to regular work in either group should, however, show in secondary outcomes, such as pain, functional status, employee satisfaction and costs.

Despite the extensive amount of quantitative data collected in this trial on individual, ergonomic, psychosocial and economic factors, we do not have the means to quantify all the aspects of the arrangements made at the workplaces during part-time sick leave. Acknowledging the potential effect of this contextual process on the outcome of the intervention, we aim to collect all relevant qualitative data during the study from the employee and the supervisor.

This is the first randomized controlled trial to our knowledge to investigate the effectiveness of early part-time sick leave in comparison to conventional full-time sick leave in musculoskeletal symptoms. The results and the increased knowledge will assist in better decision making process regarding the management of disability related to MSDs.

## Competing interests

The author(s) declare that they have no competing interests.

## Authors' contributions

EVJ conceived the idea for the study and is responsible for the protocol together with KPM, EPT, RK, HR, and JaK. RL is responsible for the statistical design of the study. KPM, HM, EVJ, LKK, and JoK have been responsible for preparing the manuscript. All authors have read and approved the final manuscript.

## Pre-publication history

The pre-publication history for this paper can be accessed here:


